# tskit_arg_visualizer: interactive plotting of ancestral recombination graphs

**DOI:** 10.1093/bioadv/vbaf302

**Published:** 2025-11-24

**Authors:** James Kitchens, Yan Wong

**Affiliations:** Department of Evolution & Ecology and Center for Population Biology, University of California—Davis, Davis, CA 95616, United States; Big Data Institute, Li Ka Shing Centre for Health Information and Discovery, University of Oxford, Oxford OX3 7LF, United Kingdom

## Abstract

**Motivation:**

Ancestral recombination graphs (ARGs) are a complete representation of the genetic relationships between recombining lineages and are of central importance in population genetics. Recent breakthroughs in simulation and inference methods have led to a surge of interest in ARGs. However, understanding how best to take advantage of the graphical structure of ARGs remains an open question for researchers. Here, we introduce tskit_arg_visualizer, a Python package for programmatically drawing ARGs using the interactive D3.js visualization library.

**Results:**

We highlight the usefulness of this visualization tool for both teaching ARG concepts and exploring ARGs inferred from empirical datasets.

**Availability and implementation:**

The latest stable version of tskit_arg_visualizer is available through the Python Package Index (https://pypi.org/project/tskit-arg-visualizer, currently v0.1.1). Documentation and the development version of the package are found on GitHub (https://github.com/kitchensjn/tskit_arg_visualizer).

## 1 Introduction

At each position in the genome, genetic relationships between sampled genomes can be described by an evolutionary (or “gene”) tree. During meiosis, recombination generates mosaic chromosomes by bringing together regions with distinct inheritance histories, such that many different local trees can exist along the chromosome. However, neighboring local trees are usually highly correlated, sharing the majority of their nodes and edges in common (Hein 1990, McVean and Cardin 2005). An “ancestral recombination graph” (ARG), first coined by Griffiths (1991), describes the set of local trees for a genomic region woven together into a graphical structure based on their shared branches [for a more complete definition of ARGs, see Hudson (1983) and Wong et al. (2024)]. ARGs capture the observable patterns of inheritance that underlie the genetic diversity of the samples (Ralph *et al.* 2020), making them an extremely data-rich object for genetic studies (Nielsen *et al.* 2025). Thanks to major advances in ARG inference methods, ARG-based analyses can now be applied to many different empirical systems (Rasmussen *et al.* 2014, Kelleher *et al.* 2019, Speidel *et al.* 2019, Ignatieva *et al.* 2021, Zhang *et al.* 2023, Deng *et al.* 2025). Coupled with the development of associated statistical methods, ARGs are currently being used to investigate a broad range of population genetics questions, including recognizing recombinant lineages (Ignatieva *et al.* 2021, Zhan *et al.* 2025), identifying selected loci (Stern *et al.* 2019, Vaughn and Nielsen 2024), and reconstructing complex demographic histories (Fan *et al.* 2025). With this growth in interest (Lewanski *et al.* 2024), there is a pressing need for tools that support ARG research and lower the barrier of entry into this field.

Visualization is critically important for teasing apart and communicating the complex relationships encoded in an ARG. This is commonly done by plotting local trees along a genome, often focusing on individual trees at loci of interest [e.g. Hubisz and Siepel (2020)], or emphasizing the correlation between trees using colors or additional lines [e.g. Rasmussen and Guo (2023)]. However, this tree-based approach necessarily obscures how these trees fit into the larger ARG. Alternatively, as an ARG is a form of directed acyclic graph, the graph structure itself can be visualized. For example, classical depictions generally show a network of nodes organized by time, linked by vertical and horizontal edges, with the root at the top and samples at the bottom (Griffiths 1991, Griffiths and Marjoram 1996, Wiuf and Hein 1999). Although useful with small, hand-built examples (Rasmussen *et al.* 2014, Lewanski *et al.* 2024), the resulting visualization can be confusingly tangled and hard to interpret for larger empirical ARGs which often reflect highly reticulated ancestries. Moreover, this visualization strategy can make it hard to focus on specific genomic loci of interest, as local trees are more difficult to discern amid the larger structure of the graph.

Here, we present tskit_arg_visualizer, a graph-based visualizer which addresses the problems of tangling and local tree representation using modern software techniques. Force-directed simulation is used to untangle the graph, and interactive highlighting is used to reveal both local trees and the extent of genomic regions spanned by edges and their associated mutations. In doing so, the visualizer draws the full ancestral structure, while simultaneously allowing the user to explore genetic relationships within local genomic regions. tskit_arg_visualizer adds to a growing list of tools aimed at teasing apart these data-rich graphs.

## 2 Results

### 2.1 Implementation

tskit_arg_visualizer is a Python package that leverages the D3.js visualization library to draw interactive ARGs in a browser-based environment. When run from the command-line, this package launches a browser window; alternatively, plotting is possible directly inside a Jupyter Notebook. Figures can also be drawn inside Quarto presentations, providing interactivity when teaching about ARGs in a class or workshop setting.

### 2.2 Model

**Figure 1.**
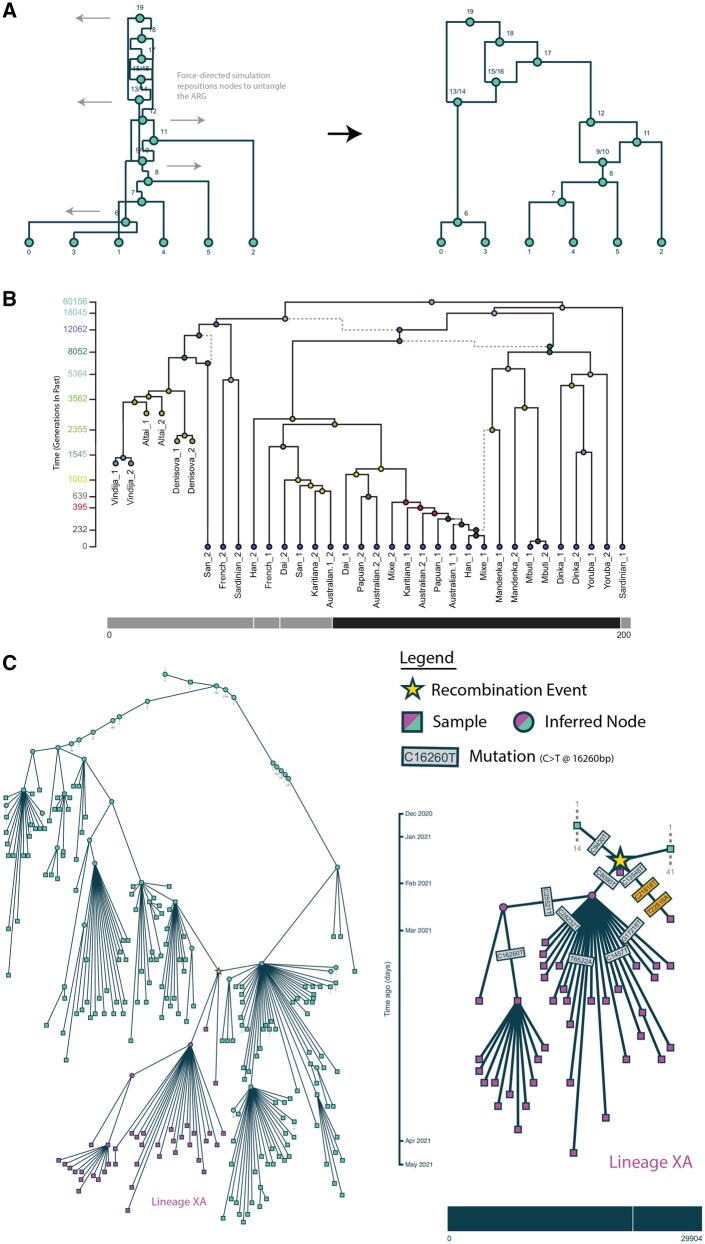
(A) The start and end layouts of a simulated ARG after nodes were repositioned by the force-directed simulation. (B) An inferred ancestral recombination graph covering 200 bps of the human genome within the Duffy antigen receptor gene (DARC). This depicts the ancestry of 14 modern humans and three ancient samples. The local tree of SNP rs2814778 (1:159174683) is marked with solid lines. Dashed lines indicate edges that do not appear in the local tree. The y-axis shows the number of generations in the past that events occurred. Nodes and edges were positioned with draw(), exported as an SVG, and further stylized using Adobe Illustrator. (C) Two SARS-CoV2 subgraphs at different zoom levels for Pangolin XA recombinant genomes. Both are created using draw_node() without further modification outside of the visualizer. The zoomed out subgraph (left) tracks the lineages above the recombination node back to samples from December 2019. Dotted lines show connections to the remaining 2.7 million ARG nodes. All 39 samples descending from the recombination node (star) are Pangolin XA recombinants. A nonlinear, ranked timescale has been used for efficient vertical node spacing. Mutations have been added to the zoomed in subgraph, and the mutations in the spike region have been plotted in gold. Further details regarding the construction and visualization of the ARGs in (B) and (C) can be found in the supplementary data at *Bioinformatics Advances* online.

As the name suggests, tskit_arg_visualizer is designed to easily integrate into the tskit ecosystem of packages and provides functions to convert from a tskit.TreeSequence in a manner that scales to millions of nodes and edges (as was required for Fig. 1C). This package represents an ARG as a collection of tables for nodes, edges, mutations, and breakpoints, altogether referred to as a D3ARG. This mirrors the tskit tree sequence tabular format but with a focus on efficiency of visualization rather than efficiency of storage. For example, in addition to graph specific details such as node times and connections, tables contain styling information used to customize the visualization. Another key difference is that an edge in a D3ARG can contain disjoint chromosomal intervals, whereas a tskit tree sequence stores a separate edge per interval. Additionally, tables can be manually built without tskit by using the pandas Python package (The Pandas Development Team 2025) or importing from a previously saved JSON file.

### 2.3 Visualizations

tskit_arg_visualizer offers two methods for viewing an ARG:

- draw(): displays the full graph to the screen (Fig. 1A and B)
- draw_node(): useful for larger ARGs, first filters to a subgraph around a focal node before displaying (Fig. 1C)

These can be combined with two edge-drawing stylizations. The conventional “orthogonal” format, shown in Fig. 1B, connects parent and child nodes via vertical and horizontal lines. It is particularly suitable for “full ARGs” (Wong *et al.* 2024) with marked recombination nodes and where common ancestor nodes have a maximum of two children. The more flexible “line” stylization, shown in Fig. 1C, connects each parent to its child via a straight line, which better accommodates ARGs containing nodes with many direct connections.

In all cases, a customized force-directed simulation is used to untangle graph edges (Eades 1984). Force-directed simulations balance a combination of “forces” to position nodes in a visually interpretable way — often where nodes repel one another but are held together by edges in the graph that act as springs. A major advantage of this algorithm is that it can limit the number of crossed lines without the need for scenario specific rules or knowledge of the graph’s shape. As the y-axis position of a node corresponds with its age, we fix this value and only allow the node’s x-axis position to be updated by the simulation. Force-directed simulations are likely to settle into local optima rather than finding the true minimum number of crossed lines, so the visualizer also allows nodes to be dragged left and right along the x-axis by the user, helping to further untangle the graph if needed.

The visualizer provides many interactive features that use the chromosome bar drawn under each graph. Hovering over a region of this bar highlights its corresponding local tree embedded in the graph. Conversely, hovering over an edge of the graph highlights the genomic regions for which that edge covers. Mutations can be displayed on edges and colored arbitrarily (Fig. 1C); hovering over a mutation highlights its position on the genome bar, and hovering over an edge similarly highlights the genomic position of all the mutations on that edge.

All figures can be saved into a variety of file formats, including PNG, SVG, and JSON. The latter can be passed back into the visualizer with draw_D3() to reproduce figures exactly.

## 3 Conclusion

tskit_arg_visualizer offers methods for programmatically plotting ARGs and provides many interactive features to help researchers explore these graphs. Visualizing ARGs, either in their entirety or by zooming in to specific substructures, can help researchers identify elements related to critical biological processes or concerns in data quality control. The visualizer has proved to be a useful tool for communicating about ARG concepts to a broader audience within the online tskit tutorials and during multiple conference workshops.

## Supplementary Material

vbaf302_Supplementary_Data

## Code availability

tskit_arg_visualizer continues to be actively updated with new features. Stable releases are published to the Python Package Index (https://pypi.org/project/tskit-arg-visualizer, currently v0.1.1). The latest development version of the package can be found on GitHub (https://github.com/kitchensjn/tskit_arg_visualizer).
