# Supplementary material for: tskit_arg_visualizer: interactive plotting of ancestral recombination graphs
Source: Bioinform Adv. 2025 Nov 24;5(1):vbaf302. doi: 10.1093/bioadv/vbaf302 (PMC12701794; doi:10.1093/bioadv/vbaf302)
Supplement: vbaf302_Supplementary_Data [file vbaf302_supplementary_data.zip › KitchensWong_2025_SupplementalMaterials.pdf]

tskit_arg_visualizer: interactive plotting of ancestral recombination graphs Supplemental Materials

James Kitchens[1] and Yan Wong[2]

[1]Department of Evolution & Ecology and Center for Population Biology, University of California - Davis
[2]Big Data Institute, Li Ka Shing Centre for Health Information and Discovery, University of Oxford

November 2025

1 Creating Figure 1

Code for each of the following subfigures can be found https://github.com/kitchensjn/tskit_arg_visualizer/docs/

1.1 A

The ARG in this figure was simulated in with msprime. The version on the left was created by preventing the force-directed simulation from updating the node positions and saving the resulting ARG as an SVG. The versino on the right was saved after the force-directed simulation had settled into an optimum.

1.2 B

Following the ARGweaver tutorial from [1], we sampled ARGs for 14 modern humans and 3 ancient samples around the DARC gene on chromosome 1. A random sampled ARG was trimmed to 100 basepairs on either side of the rs2814778 variant (1:159174583-159174783). This variant is found at high frequency in many African populations, excluding the San, and is believed to be under selection for malaria resistance [3]. The local tree for rs2814778 is very similar to that presented in Figure 5 from [1], but it is now interwoven into the context of the surrounding trees. ARGweaver groups nodes into discrete time bins; for visual clarity, we separated the nodes by rank but colored them based on their true time (shown along the y-axis). The initial force-directed simulation untangled most of the graph, although some manually node positioning was required. The plot was downloaded as an SVG and brought into Adobe

Illustrator to finish stylizing in preparation for a manuscript quality figure. For instance, though the visualizer provides a rudimentary y-axis by default, the y-axis in this figure was added post-hoc to better show the time discretization implemented by ARGweaver.

1.3 C

Figure 1C shows a small portion of a large ARG of 2,689,054 nodes, inferred using the sc2ts software [5] from 2,482,157 coronavirus samples provided by the Viridian project [2]. Importing this ARG into the `tskit_arg_visualizer` took under a minute on a laptop. We've drawn two subgraphs, each at a different zoom level. The zoomed out subgraph (left) tracks the lineages above the recombination node back to samples from December 2019. We passed multiple focal nodes into the 'draw_nodes()' function to include more clades in the subgraph. This provides general context for the position of the subgraph on the right within the larger graph. This smaller subgraph only used one focal node (the recombination event) and included the parents of the recombination event and all nodes beneath it. The figure illustrates the earliest widely accepted SARS-CoV2 recombinant, labeled XA by the Pangolin project [4], which is a combination of the B.1.177.18 lineage (to the left of the breakpoint) and the B.1.1.7 lineage (to the right). The y-axis of the plots is non-linear and sorts nodes by date but skips dates that are not observed in the subgraph. Because of this, the visualizer draws different lengths of edges between the two subgraphs, though in linear time, they would have the same length. Mutations have been plotted using the default labeling scheme, showing the ancestral allelic state plus the genome position, plus the derived state. These subgraphs were drawn completely within the visualizer.

References

[1] Melissa Hubisz and Adam Siepel. Inference of Ancestral Recombination Graphs Using ARGweaver. *Methods in Molecular Biology (Clifton, N.J.)*, 2090:231–266, 2020.

[2] Martin Hunt, Angie S. Hinrichs, Daniel Anderson, Lily Karim, Bethany L. Dearlove, Jeff Knaggs, Bede Constantinides, Philip W. Fowler, Gillian Rodger, Teresa Street, Sheila Lumley, Hermione Webster, Theo Sanderson, Christopher Ruis, Benjamin Kotzen, Nicola de Maio, Lucas N. Amenga-Etego, Dominic S. Y. Amuzu, Martin Avaro, Gordon A. Awandare, Reuben Ayivor-Djanie, Timothy Barkham, Matthew Bashton, Elizabeth M. Batty, Yaw Bediako, Denise De Belder, Estefania Benedetti, Andreas Bergthaler, Stefan A. Boers, Josefina Campos, Rosina Afua Ampomah Carr, Yuan Yi Constance Chen, Facundo Cuba, Maria Elena Dattero, Wanwisa Dejnirattisai, Alexander Dilthey, Kwabena Obeng Duedu, Lukas Endler, Ilka Engelmann, Ngiambudulu M. Francisco, Jonas Fuchs, Etienne Z. Gnimpieba,

Soraya Groc, Jones Gyamfi, Dennis Heemskerk, Torsten Houwaart, Neiyuan Hsiao, Matthew Huska, Martin Hölzer, Arash Iranzadeh, Hanna Jarva, Chandima Jeewandara, Bani Jolly, Rageema Joseph, Ravi Kant, Karrie Ko Kwan Ki, Satu Kurkela, Maija Lappalainen, Marie Lataretu, Jacob Lemieux, Chang Liu, Gathsaurie Neelika Malavige, Tapfumanei Mashe, Juthathip Mongkolsapaya, Brigitte Montes, Jose Arturo Molina Mora, Collins M. Morang'a, Bernard Mvula, Niranjan Nagarajan, Andrew Nelson, Joyce M. Ngoi, Joana Paula da Paixão, Marcus Panning, Tomas Poklepovich, Peter K. Quashie, Diyanath Ranasinghe, Mara Russo, James Emmanuel San, Nicholas D. Sanderson, Vinod Scaria, Gavin Screaton, October Michael Sessions, Tarja Sironen, Abay Sisay, Darren Smith, Teemu Smura, Piyada Supasa, Chayaporn Suphavilai, Jeremy Swann, Houriiyah Tegally, Bryan Tegomoh, Olli Vapalahti, Andreas Walker, Robert J. Wilkinson, Carolyn Williamson, Xavier Zair, IMSSC2 Laboratory Network Consortium, Tulio de Oliveira, Timothy EA Peto, Derrick Crook, Russell Corbett-Detig, and Zamin Iqbal. Addressing pandemic-wide systematic errors in the SARS-CoV-2 phylogeny, November 2024. Pages: 2024.04.29.591666 Section: New Results.

[3] Kimberly F. McManus, Angela M. Taravella, Brenna M. Henn, Carlos D. Bustamante, Martin Sikora, and Omar E. Cornejo. Population genetic analysis of the DARC locus (Duffy) reveals adaptation from standing variation associated with malaria resistance in humans. *PLOS Genetics*, 13(3):e1006560, March 2017. Publisher: Public Library of Science.

[4] Andrew Rambaut, Edward C. Holmes, Áine O'Toole, Verity Hill, John T. McCrone, Christopher Ruis, Louis du Plessis, and Oliver G. Pybus. A dynamic nomenclature proposal for SARS-CoV-2 lineages to assist genomic epidemiology. *Nature Microbiology*, 5(11):1403–1407, November 2020. Publisher: Nature Publishing Group.

[5] Shing H. Zhan, Anastasia Ignatieva, Yan Wong, Katherine Eaton, Benjamin Jeffery, Duncan S. Palmer, Carmen Lia Murall, Sarah P. Otto, and Jerome Kelleher. Towards Pandemic-Scale Ancestral Recombination Graphs of SARS-CoV-2, June 2023. Pages: 2023.06.08.544212 Section: New Results.
